# Fostering Competence in Medicines Development: The IFAPP Perspective

**DOI:** 10.3389/fphar.2016.00377

**Published:** 2016-10-14

**Authors:** Dominique J. Dubois, Anna Jurczynska, Sandor Kerpel-Fronius, Gustavo Kesselring, Kyoko Imamura, Gerfried Nell, Honorio Silva, Peter Stonier

**Affiliations:** ^1^Pharmed, Université Libre de BruxellesBrussels, Belgium; ^2^International Federation of Associations of Pharmaceutical Physicians & Pharmaceutical MedicineWoerden, Netherlands; ^3^Spanish Association of Pharmaceutical MedicineMadrid, Spain; ^4^Department of Pharmacology and Pharmacotherapy, Semmelweis UniversityBudapest, Hungary; ^5^Medscience Clinical Research & Medical Affairs ConsultingSão Paulo, Brazil; ^6^Office of Pharmaceutical MedicineYokohama, Japan; ^7^NPC Nell Pharma Connect LtdWien, Austria; ^8^Rutgers University School of Health Related ProfessionsNewark, NJ, USA; ^9^Institute of Pharmaceutical Sciences, King's College LondonLondon, UK

**Keywords:** competencies, certification, patient outcomes, medical affairs, outcomes based education, patient centricity, ethics

## Abstract

IFAPP (International Federation of Associations of Pharmaceutical Physicians and Pharmaceutical Medicine) is a nonprofit organization with the mission to promote Pharmaceutical Medicine & Medicines Development (PM&MD) by enhancing the competencies and maintaining high research ethical standards of Pharmaceutical Physicians and other professionals involved in medicines development worldwide, leading to the availability and appropriate use of medicines for the benefit of patients and society^1^. About 30 national professional associations related to PM&MD, involving 7000 professionals, are affiliated to IFAPP. Medicines development has traditionally been a challenging enterprise, with high risk, high investment, and potentially high returns in the lengthy and complex process of identifying a new chemical entity as a candidate for development and possibly succeeding in bringing it as a pharmaceutical product to the market. However, the emergence of genomics, translational research, biomarkers, and precision medicine pose challenges going forward involving allocation of resources, price, market access, and cost-effectiveness as opposed to the traditional concepts of “efficacy” and “safety.” Education and Continuing Professional Development (CPD) are a major focus of IFAPP. The International Conference on Pharmaceutical Medicine (ICPM) is the largest event for our organization; ICPM is held every 2 or 3 years and is aimed to provide the state of the art in key areas for our discipline and profession. The paper is a reflection on the role of competency-based education and training for Pharmaceutical Physicians and medicines development scientists, as was discussed during the recent ICPM 2016 held in Sao Paulo, Brazil on April 18–19, with the support of the Brazilian Association of Pharmaceutical Medicine, and gathered around 200 representatives from the pharmaceutical, clinical research and regulatory arenas from all over the world^2^^,^^3^.

## Introduction

Pharmaceutical medicine is defined by the Faculty of Pharmaceutical Medicine as “the medical scientific discipline concerned with the discovery, development, evaluation, registration, monitoring, and medical aspects of marketing of medicines for the benefit of patients and the health of the community^4^.”

The discipline involves several traditional professions and activities, such as medicine, pharmacy, clinical pharmacology, pharmaceutical sciences biology, etc. Therefore, the terms Pharmaceutical Physicians (PPs) and Medicines Development Scientists (MDS) refer to individuals with different academic backgrounds working in similar fields.

The latter term refers to experts who are not medically qualified, work in various fields of natural sciences, pharmacy, and medical device engineering, have adequate training in non-clinical and clinical aspects of medicines development and work as integrated members of clinical medicines development teams. Pharmaceutical Medicine and Medicines Development are thus evolving into integrated concepts.

“At core of the discipline is: the clinical testing of medicines, translation of pharmaceutical drug research into new medicines, safety and well-being of research participants in clinical trials, understanding the safety profile of medicines and their benefit-risk balance^4^.”

These are the core domains of Pharmaceutical Medicine and Medicines Development. “In addition to expertise in the science of drug development PPs and MDS need a thorough understanding of pharmacoeconomics, medical aspects of the marketing of medicines, medical affairs, business administration, and the social impact of healthcare on patients and public health. PPs and MDS work in the pharmaceutical industry, drug regulatory authorities and contract research organizations, but have a close affinity with their medical colleagues in primary and secondary health care and at universities^4^.”

Two recent trends—one in drug development and one in drug regulation—are reinforcing the importance of earlier drug evaluation and the conduct of clinical trials. In recent years the pharmaceutical industry has showed a growing interest in developing drugs for niche markets. The narrow population base for these therapies often inherently limits the amount of safety and efficacy data available to support the traditional regulatory approval, which underscores the importance of assessing the benefits and risks of these new drugs through the ongoing collection and analysis of post-marketing surveillance data and comparative effectiveness studies. The criteria for efficacy and safety of new medicines are evolving continuously and are discussed regularly in scientific fora.

As a professional organization IFAPP has been actively involved in creating awareness and fostering education and training in the following areas: Competencies and Competency Based Education; Patient Centricity and Patient-Reported Outcomes Measures (PROMs); Certification and Specialization; the emerging role of Medical Affairs and the need for an ethical framework in the practice of Pharmaceutical Medicine.

## Competencies and competency based education

Medicines Development has become a compartmentalized and segmented activity and gradually the focus is on education and training for the individual compartments or domains described above (safety, regulatory, clinical research, medical affairs, health outcomes, etc). However, the principles of Pharmaceutical Medicine as a discipline and a professional activity are fundamental to ensure a proper balance in meeting the needs of the health-care system, the patient, the regulatory agencies, and the pharmaceutical industry. Therefore, a professional involved in medicines development should be able to master the competencies needed for an effective performance.

Competencies refer to the “observable ability of a health professional integrating knowledge, skills, values and attitudes” to perform effectively. Competencies are the ingredients of Competence. A competent professional is the one possessing the required abilities (competencies) in all domains in a certain context at a defined stage of education or practice. The progression of competence, from novice to mastery, can be also defined (Frank et al., 2010).

IFAPP and PharmaTrain created a working group including representatives from either institution, with special interest and experience on quality improvement through education. As a result a basic set of 7 domains and 57 competencies and a Statement of Competence were defined (Silva et al., 2013, Table 1). The competencies were aligned with the learning outcomes of the base course offered by PharmaTrain^5^ and thus a standard for competency-based education (at the cognitive level) could be used for certification of the IFAPP membership.

**Table 1:** Statement of Competence^*^.

**STATEMENT OF COMPETENCE**
**The Pharmaceutical Physician/Medicines Development Scientist:**
• Is able to identify unmet therapeutic needs, evaluate the evidence for a new candidate for clinical development and design a Clinical Development Plan for a Target Product Profile.
• Is able to design, execute and evaluate exploratory and confirmatory clinical trials and prepare manuscripts or reports for publication and regulatory submissions.
• Is able to interpret effectively the regulatory requirements for the clinical development of a new drug throughout the product life-cycle to ensure its appropriate therapeutic use and proper risk management.
• Is able to evaluate the choice, application and analysis of post-authorization surveillance methods to meet the requirements of national/international agencies for proper information and risk minimization to patients and clinical trial participants.
• Is able to combine the principles of clinical research and business ethics for the conduct of clinical trials and commercial operations within the organization.
• Is able to appraise the pharmaceutical business activities in the healthcare environment to ensure that they remain appropriate, ethical and legal to keep the welfare of patients and research participants at the forefront of decision-making in the promotion of medicines and design of clinical trials.
• Is able to interpret the principles and practices of people management and leadership, using effective communication techniques and interpersonal skills to influence key stakeholders and achieve the scientific and business objectives.

* Adapted from Silva et al. (2013).

More recently, another working group developed the full set of applied knowledge, skills, behaviors and attitudes for each competency and a process for internal and external validation was initiated^6^. The full core competencies are the foundation for the curriculum of the Specialist in Medicines Development, one of the certification programs developed in collaboration with PharmaTrain.

IFAPP representatives have also been involved in the definition and validation of core competencies in clinical research (Sonstein et al., 2014, in press).

The competencies are intended to be used as a resource and guide to improve the quality and accountability of Pharmaceutical Medicine education and training (Sonstein et al., 2014, Table 2). The model may foster further granularity and thus specific sub-competencies and specialty competencies that apply to specific functions in clinical research and drug development could be identified. The primary vision for this competency model is the availability of professionals more fully prepared for the many challenges and opportunities in Pharmaceutical Medicine in the next decade.

**Table 2:** Potential Use of Professional Competencies in the creation of standards^*^.

• Curriculum development
• Training initiatives
• Basic training requirements
• Guidance for job descriptions and job portfolios
• Defining professional careers and pathways
• Performance evaluations
• Policy Development
• Regulatory Compliance
• Quality Improvement
• Accreditation of academic programs
• Professional Certification
• Performance across the medicines development chain

* Adapted from Sonstein et al. (2014).

We intend to use the core competencies to define profiles of key functions in medicines development. Standardized job descriptions for various roles could be developed globally to reassure every stakeholder that the processes related to medicines development are in the hands of competent biomedical professionals.

Competency models are iterative processes, and our model will have to be regularly updated as the competencies are deployed and used for professional, academic or self-assessment purposes and the business and scientific environment change.

## The example of patient-reported outcomes measurement

The use of Patient-Reported Outcomes Measures (PROMs) is a growing emerging trend and challenge in clinical research. PROMs are particularly important in clinical trials because they are the only measure that directly reflects the patient's own perspective on the impact of treatment on health and disease. Measuring the impact of new treatments on Health-Related Quality of Life (HRQL) in clinical trials is particularly important, adding a unique and highly relevant perspective on efficacy as perceived by the patient (Acquadro et al., 2003).

Thanks to an increasing interest in the patient perspective, HRQL questionnaires are nowadays frequently used and considered important by investigators, registration bodies, health authorities, and health technology assessment officers.

Disease specific questionnaires for new treatment indications and/or orphan diseases are the ones that create a problem given the financial hurdles and proprietary issues related to new assessment tools. Small start-up companies might be particularly affected because of their limited resources and expertise in HRQL matters.

Measuring HRQL is complex. As recommended by the FDA, the first step in the selection of a HRQL measurement instrument is to develop a conceptual framework based on the concepts of interest for measuring meaningful treatment benefit to the patient, and to determine the intended population for the clinical trial. The second step consists of developing an endpoint model identifying the relationship among clinical and patient-based outcomes of interest^7^. This will provide the basis for identifying or adapting available validated questionnaires that measure the concept(s) of interest in the context of use. If no suitable questionnaires are identified, for example in a new therapeutic area, it may be necessary to develop a new questionnaire, adding to the complexity, time, and cost of including HRQL measures.

Clinical trials involving patients with rare diseases are particularly challenging for the selection, implementation, and interpretation of PROMs. This is mainly due to the small number of patients usually included and the high heterogeneity of the patient populations^8^.

The increasing use of electronic versions of PRO questionnaires instead of paper and pencil administration may help to reduce the time spent for questionnaire completion and data collection, but adds further to the cost and complexity of validating this mode of administration. The linguistic validation procedures for demonstrating cross-cultural validity add further complexity when including PROs in multi-country studies.

Unfortunately, HRQL assessments are often of poor quality (Martini et al., 2016), despite many guidelines^9^^,^^10^. In the domain of cancer, this has led the American Society of Clinical Oncology (ASCO) to excluding HRQL from its conceptual framework to assess the value of new options for cancer treatment (Schnipper et al., 2015, 2016)^11^.

The importance of abiding by scientific methods and guidelines in conducting and reporting HRQL studies and the increasing complexity of selecting and developing new questionnaires has prompted IFAPP to include HRQL in the Competence in Medicines Development topics which would serve as the basis to develop specific education and training programs aimed to foster the proper application of guidelines and publishing PRO results.

## Fostering the emerging role of medical affairs

Medical Affairs organizations have emerged over the past half century in response to federal regulations around the separation of medical and commercial activities within drug companies. They aim to provide patient and physician centered services as part of a new business model aimed to provide value in healthcare. Many companies also chose to focus R&D resources on developing new products and moved post-launch activities, such as finding new indications for existing drugs, into the Medical Affairs function.

Continued pressures from regulatory agencies and public sentiment have pushed more and more activities into Medical Affairs organizations. Today, these organizations commonly involve the following medical activities:

- Medical education
- Medical field teams
- Post-launch clinical trials
- Medical-information services
- Medical communications
- Medical strategic activities
- Medical grants
- Publications
- Health Economics and Outcomes Research

These functions make relevant contributions in the decision making process among key medical stakeholders and customers by facilitating coordination and integration of medical data and knowledge.

A special session related to “The future of Medical Affairs organizations in pharmaceutical companies” was held during ICPM 2016 and key executives from the Medical Departments from major pharmaceutical companies were invited to discuss the needs for the development and evolution of Medical Affairs departments within pharmaceuticals, to share their vision and expectations as senior medical leaders for their organizations in order to provide patient and physician centered services for improved health care and to discuss the challenges and opportunities to leveraging the reputation and credibility of the industry in public opinion.

Panelists discussed and concurred that physicians and other professionals who serve in the Medical Affairs areas are positioned at a dynamic and critically important interface between the biopharmaceutical industry and the medical community. Their role is to communicate data-driven findings to the medical world while obtaining input from physicians and leaders in medicine about clinical issues and the direction that medicine is taking. They also highlighted that the medical interface between a healthcare company and the practice of medicine—the physician-to-physician interface with a culture based on the proper practice of medicine is at the root of a successful medical affairs organization. All these companies confirmed that their Medical Affairs organization consists of individuals who take an external focus, provide an outside-in perspective, and possess the ability to influence internally and has a growing importance in places where the role of the physician is evolving in healthcare (away from business/purchasing focus and into advocacy as part of larger systems).

As an outlook for the future, the panelists agree many companies are fostering the creation and further development of Medical Affairs Units in their structure, aiming to strengthen the peer-to-peer communication and building credible expertise in local healthcare networks/systems instead of the usual commercial approach of just “promotion of” pharmaceuticals. Several examples of biased medical information have been cited in the past few years. The withholding of product information that may affect the overall profile of efficacy and safety for any product may have a significant negative impact among all stakeholders, particularly patients, and the regulatory agencies, and affects the overall reputation of the industry (Cohen, 2014)^12^.

Critically important to the success and contributions for the future of Medical Affairs organizations will be the proper training in the sciences fundamental to medicine and the principles of Medical Affairs as a discipline. There was consensus that education and training of biomedical personnel have become a critical need within pharmaceuticals and therefore competent professionals are needed.

## Professional certification and specialization

*Professional certification*, often called simply *certification* or *qualification*, is a designation earned by a person to assure qualification to perform a job or task. Certification does not refer to the state of legally being able to practice or work in a profession. That is *licensure.* Usually, licensure is administered by a governmental entity for public protection purposes and a professional association administers certification. Licensure and certification are similar in that they both require the demonstration of a certain level of knowledge or ability. However, certification is a pre-requisite for licensure. *Specialization* is a type of certification, usually required for licensure.

Although fostering the development and international recognition of Pharmaceutical Medicine as a separate medical specialty has been one of the key goals for IFAPP since its inception, the objective has been only partially met. A number of possible explanations (not enough advocates, lack of awareness on the discipline among the country decision makers, no established need for licensure, limited recognition to new medical specialties at the country level, etc.) can be attributed to such lack of success.

Since the certification is granted by the profession, the appropriate professional certifying bodies would be IFAPP and PharmaTrain, and because of their global nature would provide the required international scope.

IFAPP (in collaboration with PharmaTrain and academic institutions) is entitled to provide professional certification to its membership, in a two-step process as separate programs to attest the new competency-based standards for professionals involved in medicines development. A basic certification (based upon knowledge) and a vocational certification (based in competencies) are being developed.

### Knowledge based certification

This is the theoretical/cognitive competency part of the SMD program, offered via online CPD in strategic alliance with academic institutions. The cognitive contents are included in 6 modules, with a high level of interactivity, which can be completed in a 6–12 month period. Assessments will be conducted at the end of each module and a *Certificate on Medical Affairs and Clinical Development* should be granted to students completing the 6 modules. The production of the e-learning content is planned to be completed in 2016 so that the Certification of the IFAPP membership may start in 2017 and the first cohort of certified professionals can be expected in 2018.

### Vocational specialization program

The Specialist in Medicines Development (SMD) is a competency-based workplace-centered 2- to 4-year education and training program, comprising a knowledge based covering the PharmaTrain syllabus achieved through IFAPP/PharmaTrain accredited graduate programs in Pharmaceutical Medicine or its equivalent (the above mentioned Certificate Program on Medical Affairs and Clinical Development). Participants in this mentored program acquire competencies within a frame work of assessment, appraisal, and annual review of progress and achievement. On completion, participants achieve the Specialist in Medicines Development awarded by both IFAPP and PharmaTrain. Pilot experiences have started in Italy and Japan.

IFAPP will continue its efforts to foster Pharmaceutical Medicine/Medicines Development as an independent scientific medical discipline and as a viable and rewarding professional path for biomedical graduates. It is our goal for the coming years.

## Ethical framework for professionals involved in medicines development

The new technologies, molecular biology, translation medicine, advanced therapies, medical device-drug combinations, etc. made the development of medicines much more complex. The traditional approach focusing almost exclusively on the role of the physicians having a combined training of pharmacology and therapeutics, frequently referred to as PPs or clinical pharmacologists in the different countries, was gradually replaced by complex clinical investigator teams. Many of these technologies can be applied only by basic scientists who frequently are not medically qualified. Nevertheless, their role can be crucial in making decisions critically influencing the conduct of studies and clinical trials, and the well-being of the volunteer research participants. These changes made it necessary to reconsider the ethical concepts underlying clinical trials. The ethical ideas formulated by the Faculty of Pharmaceutical Medicine of the Royal Colleges of Physicians of the UK (The Faculty) and those by IFAPP were presented at the ICPM 2016 plenary session entitled “Ethics in Medicines Development: where we stand and were we go.” It is interesting to note that the two sets of ethical recommendations are conceptually different, nevertheless, mutually supportive. The target audience of the Faculty recommendations are the PPs whilst the IFAPP recommendations consider equally the PPs as well as the MDS involved.

The Faculty document entitled Good Pharmaceutical Medical Practice (GPMP) published on 12th November 2014 is essentially an expanded version of the UK General Medical Council's Good Medical Practice^13^. The Faculty's intention is to use GPMP as an adjunct to GMP in the revalidation process of PPs working in the United Kingdom. Nevertheless, in the introduction of the document the authors stated that the recommendations “provide all doctors around the world specific guidance and direction on expected standards, conduct and behavior” in drug development. Since addressees of the document are exclusively PPs it is only briefly mentioned that decisions are frequently made by a team and the PPs must be prepared to explain and justify their specific contribution to decisions that may affect the safety and well-being of patients. A GPMP Support Network was created for advising PPs facing ethical or medical dilemmas in their work.

IFAPP published its International Code of Ethical Conduct for PPs in 2003^14^^,^^15^. Since that time, the membership of IFAPP has undergone major changes due to the influx of many basic scientists. These scientifically qualified experts make up now close to half of the membership. It was recognized that MDS play increasingly important roles not only by providing scientific and methodological expertise for the projects but also as active decision making members of the clinical investigator teams. Therefore, it was decided to reconsider the guiding concept of the original document and address the ethical issues jointly of both the PPs and the MDS. Accordingly, the title of the document was also changed to “IFAPP International Ethics Framework for PPs and Medicines Development Scientists^16^.”

It is argued that due to the joint participation of PPs and MDS in decision making concerning for example the selection of appropriate biomarkers, laboratory methods, the determination of the amount of biological sample needed, the production of personalized biological, or advanced medicines, etc., the ethical responsibilities have to be shared. In addition, it must be ensured that all the investigations applied in the development of medicines should be carried out according to the strict quality standards accepted for the clinical development of medicines which are more stringent than those traditionally accepted in basic research. In spite of the close cooperation and joint ethical responsibilities, it is emphasized that the supervision of the well-being of the clinical trial participants remains the exclusive responsibility of a qualified physician participating in the development team. The situation is, however, quite complex since the differently trained members of the development team might have different ethical priorities under various situations. Therefore, it was considered to be inappropriate to define strict ethical guidelines. It was the firm belief of the revising committee that a framework of advices on correct ethical behaviors and appropriate actions under selected circumstances is a more practical support for teams developing complex medicines.

During the discussion it was emphasized that the two documents should be used jointly since they emphasize different but equally important aspects of modern medicines development. It is planned to publish the two documents side by side, thus underlying the need for their joint application in medicines development.

## Future directions

As a professional organization IFAPP is committed to foster the development of key areas for the discipline of Pharmaceutical Medicine and the profession of Medicines Development in order to ensure patients and the society at large that the medicines development process is conducted by competent individuals.

Competency-based education is now a paradigm for undergraduate, postgraduate and continuing professional development and poses another continuing challenge to the academic institutions and professional associations for development and assessment of education and training programs.

## Author contributions

Each author has been sufficiently involved in this submission to take public responsibility for the work, meaning that each author has made substantial contributions to the conception and design of the study (with further contributions by DD, PS, HS, SK, and GK), or acquisition of data (with further contributions by DD, HS, SK and PS), or analysis and interpretation of the data (PS, HS, DD), and drafting the article and revising it critically for important intellectual content (HS, PS, DD, GK). All authors read and approved the final manuscript.

### Conflict of interest statement

The authors declare that the research was conducted in the absence of any commercial or financial relationships that could be construed as a potential conflict of interest.

## Footnotes

^4^*Adapted from “What is Pharmaceutical Medicine?”* Available at: https://www.fpm.org.uk/aboutus/whatispharmamed (Accessed latest; 22 September, 2016).

^5^PharmaTrain. Available at: https://www.imi.europa.eu/content/pharmatrain (Accessed latest; 22 September, 2016).

^6^IFAPP: Core Competencies in Pharmaceutical Medicine and Medicines Development. Available at: http://ifapp.org/Education/Publication-on-education (Accessed latest; 22 September, 2016).

^7^FDA Clinical Outcome Assessment Qualification Program. Available at: http://www.fda.gov/drugs/developmentapprovalprocess/drugdevelopmenttoolsqualificationprogram/ucm284077.htm (Accessed latest; 22 September 2016).

^8^Clinical Outcome Assessment (COA) in Rare Disease Clinical Trials - Emerging Good Practices: Report of the ISPOR Rare Disease Trials COA Measurement Task Force. Available at: http://www.ispor.org/TaskForces/COA_Measurement_RareDiseases_2016DC_forum.pdf (Accessed latest; 22 September, 2016).

^9^EMEA Reflection paper on the regulatory guidance for the use of Health-Related Quality of Life (HRQL) measures in the evaluation of medicinal products. Available at: http://www.ema.europa.eu/docs/en_GB/document_library/Scientific_guideline/2009/09/WC500003637.pdf (Accessed latest; 21 September, 2016).

^10^EMA Appendix 2 to the guideline on the evaluation of anticancer medicinal products in man. The use of patient-reported outcome (PRO) measures in oncology studies. Available at: http://www.ema.europa.eu/docs/en_GB/document_library/Other/2016/04/WC500205159.pdf (Accessed latest; 21 September, 2016).

^11^ASCO Value Framework Update Statement by ASCO President Julie M. Vose, MD, MBA, FASCO, May 31, 2016. Available at: http://www.asco.org/advocacy-policy/asco-in-action/asco-value-framework-update (Accessed latest; 17 August, 2016).

^12^Dave D. Effects of Pharmaceutical Promotion. A review and assessment. NBER Working Paper NO. 18830. February 2013. Available at: http://www.nber.org/papers/w18830 (Accessed latest; 21 September, 2016).

^13^Faculty of Pharmaceutical Medicine. Good Pharmaceutical Medical Practice (GPMP). Available at: https://www.fpm.org.uk/policypublications/GPMP (Accessed latest; 21 September, 2016).

^14^International Federation of Associations of Pharmaceutical Physicians (IFAPP) International Code of Ethical Conduct for Pharmaceutical Physicians. Available at: http://ifapp.org/upload/file/Final-Ethics-Code-April-2003.pdf (Accessed latest; 21 September, 2016).

^15^Becker S, Barrett J, Botha JJ, Caird D, Carlesi R, Jekunen A on behalf of the IFAPP Working Party on Ethics in Pharmaceutical Medicine. A Proposed International Code of Ethical Conduct for Pharmaceutical Physicians. Available at: http://ifapp.org/upload/file/finalpostercancun-s_becker.pdf (Accessed latest; 21 September, 2016).

^16^IFAPP International Ethics Framework for Pharmaceutical Physicians and Medicines Development Scientists.” Available at: http://ifapp.org/Ethics (Accessed latest; 22 September, 2016).
